# Efficacy of combined traditional Chinese medicine spray with premature ejaculation desensitization therapy for the treatment of primary premature ejaculation

**DOI:** 10.4314/ahs.v17i3.2

**Published:** 2017-09

**Authors:** Ying-Dong Cui, Shu-Bin Hu, Bo Wu, Shi-Jun Li, Kui Xiang, Zhao-Lin Liao, Hui-Ping Zhang, Chang-Hong Zhu, Meng Rao

**Affiliations:** 1 Department of Urology, Enshi Tujia and Miao Autonomous Prefecture nationality Hospital, Enshi, Hubei, China; 2 Family Planning Research Institute, Tongji Medical College, Huazhong University of Science and Technology, Wuhan, Hubei, China

**Keywords:** Primary premature ejaculation (PPE), traditional Chinese mdicine spray (TCMS), premature ejaculation desensitization training therapy (PEDT), Intravaginal ejaculation latency time (IELT), Chinese index of sexual function for premature ejaculation (CIPE-5)

## Abstract

**Objectives:**

We recommend a new kind of spray made from eight kinds of traditional Chinese medicine, we aimed to investigate the safety and clinical efficacy of combined traditional Chinese medicine spray (TCMS) with premature ejaculation desensitization therapy (PEDT) for the treatment of primary premature ejaculation (PPE).

**Methods:**

A total of 90 patients with PPE were randomly assigned to receive TCMS, PEDT monotherapy or TCMS plus PEDT combination therapy for 6 weeks. Intravaginal ejaculation latency time (IELT) and Chinese index of sexual function for premature ejaculation (CIPE-5) were measured to evaluate the effect of each treatment.

**Results:**

Eighty six (86) participants completed the study voluntarily. Both IELT and CIPE-5 in these three groups increased after treatment when compared with baseline levels (p< 0.01). IELT and CIPE-5 after treatment in TCMS plus PEDT group were significantly higher than those in the other two groups (both p <0.05). Additionally, clinical efficacy in TCMS plus PEDT group (89.7%) was significantly higher than in TCMS (65.5%) and PEDT group (67.9%) (p< 0.01).

**Conclusion:**

The self-made TCMS was safe and effective for the treatment of PPE, a combination of TCMS and PEDT therapy was more effective than the TCMS or PEDT monotherapy.

## Introduction

Premature ejaculation (PE) is a very common male sexual dysfunction with prevalence rates of 20–30%^1^,^2^. As people's awareness on sexual attitudes changes, more attention has been paid to the impact on quality of life for patients and their sexual partners with PE. Both the patients and their partners complain about decreased sexual self-confidence and self-esteem and an overall reduction in their quality of life^3^.

PE is classified as primary (lifelong) or secondary (acquired) if it is present at almost every intercourse from the first sexual encounter on wards. While the pathophysiology of primary premature ejaculation (PPE) is not fully understood, nowadays it is clear that both organic and psychosocial factors play a role in the etiology^4^, glans penile hypersensitivity and hyperexcitability is the general reason for PPE^5^–^7^. Studies found that patients with PPE have more dorsal nerves of the penis than healthy adults, this abnormal distribution of dorsal nerves possibly leads to glans penile hypersensitivity, lowering the threshold value as well as shortening the ejaculation latency time^8^. They also generally have other abnormal autonomic reflex pathways for the ejaculatory process, including shorter bulbocavernosal latency time and higher bulbocavernosal evoked potentials^6^,^9^,^10^. Thus therapy of reducing glans penile hypersensitivity should be effective for the treatment of PPE.

Herbs have long been used in the traditional Chinese and Indian systems of medicine for the treatment of sexual dysfunction in men^11^,^12^. Many kinds of herbs have the effect of local anesthesia, vaso-activation and so on. *Radix Aconiti (Chuanwu)* was used for the treatment of a “peripheral uncomfortable feeling of cold” in Japan^13^, its active ingredients could affect peripheral vascular function via the nitric oxide (NO) system^14^. Studies showed that nitric oxide (NO) might be a neurotransmitter involved in the central and peripheral control of ejaculation^15^,^16^. *Herba Asari (Xixin)* is a traditional herb medicine used as remedies for aphthous stomatitis, toothache, gingivitis and as a local anesthetic agent in Korea and China^17^. *Pericarpium Zanthoxyli (Chuanjiao)* is a kind of medicinal and edible herb, the component of volatile oil has a strong effect of local anesthesia^18^. *Syzygium aromaticum (Dingxiang)* is not only used in China to treat male sexual dysfunction, but also used in India as an aphrodisiac to treat erectile dysfunction, the active ingredients may have ROCK-II inhibitory potential which can result in relax of corpus cavernosum, thus it is effective for sexual dysfunction^12^. Some of the above traditional herbs have been studied for the treatment of PE, others have not, we united eight kinds of traditional Chinese medicine (4 mentioned above, other 4 not mentioned) according to traditional Chinese medicine theory, and we aimed to evaluate the safety and clinical efficacy of the ethanol extract from the eight kinds of medicine for the treatment of PPE.

Because PE involves both psychosocial and physiologic determinants, a combination psychosocial and pharmacologic therapy may be the best choice to optimize efficacy and minimize relapse^19^,^20^. Behavioral therapy is the main content of psychosocial treatment. Behavioral strategies mainly include the “stop-start programme developed by Semans^21^, and its modification, the “squeeze” technique, proposed by Masters and Johnson^22^ (several modifications exist), however, it needs the partner's cooperation for a long time and its difficult on a large degree^23^. Domestic scholars reported that Weili Multifunctional Andrologic Disease Diagnosis And Treatment workstation (WLZZ-9999, made by weili new century science & tech. Co. Ltd, Beijing, China) can obtain a good effect of treatment for PE with desensitization training therapy^24^. The automated machine can help patients complete the behavioral therapy instead of the patients and their partners themselves. The course of the treatment is as follows: first, the machine gives a small stimulation to the patient's penis to the point just before ejaculation, and then stops. Once the sensation has subsided, then starts again. When the patient develops relatively longer time of ejaculation control, stronger intensity of stimulation is given to the penis gradually, then the above treatment is repeated. Generally, treatment is given 3 times a week, six times as a course of treatment.

In summary, traditional medicine has long been used to treat sexual dysfunction in many countries in Asia and should be fully exploited. In this study, we united eight kinds of traditional Chinese medicine together and made them into a spray, and explored the safety and clinical efficacy of this traditional Chinese medicine spray (TCMS) and combined TCMS with premature ejaculation desensitization therapy (PEDT) for the treatment of PPE.

## Materials and methods

### Study population

A total of 90 heterosexual men complaining of PPE were recruited from the out-patient clinic of Enshi Tujia and Miao Autonomous Prefecture nationality Hospital Department of Urology, Enshi, China, from April 2012 to April 2013. After completing description of the study to the volunteers, the written informed consent including necessary details of the study was obtained from each patient. The study protocol was approved by the Ethics Committee of Enshi Tujia and Miao Autonomous Prefecture central Hospital. The enrolled men aged 21–38 years had been in a stable, monogamous, heterosexual relationship with regular sexual intercourse at least twice per week with a cooperative female partner. All male patients were asked to avoid condom use. Demographic data of all the patients were recorded. The study was performed for 6 weeks.

### Inclusion criteria

All the patients were diagnosed according to The Diagnostic and Statistical Manual of Mental Disorders, Fourth Edition criteria of PE^25^, the condition's onset is from the first sexual experience and remains a problem throughout life, IELT was shorter than 2 min, CIPE-5 was lower than 18 scores; older than 18 years, external genitalia was normally developed, no phimosis, no obvious prepuce, and normal erectile function; receiving clinical observation and completing the trial voluntarily; blood testosterone (T), follicle stimulating hormone (FSH), luteinizing hormone (LH), prolactin (PRL) and estradiol (E2) were normal.

### Exclusion criteria

Exclusion criteria included patients with secondary PE; sexual dysfunction (decreased sexual interest, erectile dysfunction, painful intercourse, urinary tract infection, or female sexual dysfunction that affected the sexual relationship); those suffering from prostatitis; history of a psychiatric or neurological disorder; abuse (alcohol or drug); previous genitourinary system trauma or surgery; spinal cord injury; or currently taking a drug known to affect sexual function, including either topical penile applications or systemic drugs.

### Study design

All of the 90 patients were randomly divided through a simple (complete) randomization process into three groups, each consisting of 30 patients. Patients in TCMS group were given TCMS only. The TCMS was made by the hospital pharmacy department and consisted of eight kinds of traditional Chinese medicine. The manufacture method was as follows: 30 g of *Herba Asari*, 30 g of *Syzygium aromaticum*, 20 g of *Ootheca Mantidis*, 20 g of *Radix Aconiti*, 20 g of *Galla Chinensi*, 20 g of *Fructus Rosae Laevigatae*, 20 g of *Fructus Rubi*, 1 g of *Pericarpium Zanthoxyli* were accurately weighed and soaked in 100 milliliter of alcohol (concentration 95%) for 30 days, then transferred the supernatant to the watering can for standby use. (dry weight ratio of each medicine was showed in table 1). Patients were guided to spray the TCMS evenly on the glans penis surface, coronary sulcus, foreskin frenum and the surrounding area once a day. It should also been applied to the glans penis 30 min before intercourse and washed off immediately prior to coitus. Patients in PEDT group were given PEDT by the use of Weili Multifunctional Andrologic Disease Diagnosis And Treatment workstation (WLZZ-9999), 3 times a week. Patients in TCMS plus PEDT group were given combined TCMS (once a day, as well as before sexual intercourse) with PEDT (3 times a week). All of the patients were asked not to use other therapies during the 6-week treatment period.

**Table 1:** The dry weight ratio of herbal medicine in the spray.

Chinese name	Pharmaceutical name (medical parts)	Ratio
Xixin	*Herba Asari* (Dried root of *Herba Asari*)	30
Dingxiang	*Syzygium aromaticum* (Dried flower bud of *Syzygium aromaticum*)	30
Chuanwu	*Radix Aconiti* (Dried root of *Radix Aconiti*)	20
Wubeizi	*Galla Chinensis* (Dred fruit of *Galla Chinensis*)	20
Jinyingzi	*Fructus Rosae Laevigatae* (Dred fruit of *Fructus Rosae Laevigatae*)	20
Fupenzi	*Fructus Rubi* (Dred fruit of *Fructus Rubi*)	20
Sangpiaoxiao	*Ootheca Mantidis* (Dried Ootheca of mantis)	20
Chuanjiao	*Pericarpium Zanthoxyli* (Dred fruit of *Pericarpium Zanthoxyli*)	1

Net weight 161 g of herbal medicine was extracted with 100 ml (95%) alcohol for 30 days, then filtrated for standby use.

### Measurements

#### IELT

The duration of IELT is the time from penetration (vaginal penetration) until ejaculation (release of semen) and was timed on a stopwatch by ‘start’ (penetration) to ‘stop’ (ejaculation). Either of the partners was allowed to handle the stopwatch; however, it was requested that the same person remain responsible for every IELT measurement for the duration of the study, and they were asked to be honest in recording the time. They were instructed to calculate and record the exact time after ejaculation. The IELT was recorded before and 6 weeks after the treatment.

#### CIPE-5

CIPE-5 contains 5 questionnaires: IELT; difficulty in prolonging sexual intercourse; sexual satisfaction; partner's sexual satisfaction; frequency of feeling anxious, depressed or stressed in sexual activity. Each of the questions was designed to be answered by the subject on a 5 point Likert - type scale, generating each item scores as a total score. If the IELT of the patient was more than 3 minutes, the score of IELT was 5 point. The CIPE-5 was recorded before and 6 weeks after the treatment.

#### Clinical efficacy

Clinical efficacy was compared with the prolongation of IELT and elevation of CIPE-5 scores before and after each treatment. If the prolongation of IELT was more than 2 minutes as well as the elevation of CIPE-5 was higher than 18 scores, the trial was considered effective, on the contrary, the trial was considered invalid. Efficacy rate was calculated in each group.

### Statistical analysis

#### Descriptive data were presented as mean± SD (standard deviation).

Demographic data, IELT, CIPE levels among each group were compared using Kruskal-Wallis test, comparisons before and after treatment in each group were also performed using Kruskal-Wallis test, due to skewness in the distributions of some of these measurements. Efficacy rate among each groups were compared with chi-square test. All statistical analysis was performed using the spss 17.0 software. P<0.05 was considered statistically significant.

## Results

### Demographic data, IELT and CIPE-5 baseline.

**Figure 1:**
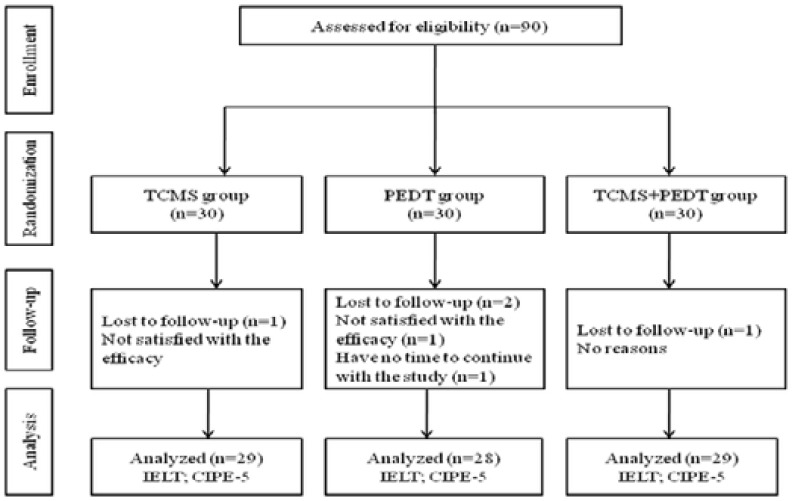
Flow chart of the study.

Of all the 90 enrolled patients, 4 withdrew from the study (1 from TCMS group, 2 from PEDT group and 1 from combination group). So a total of 86 patients completed the study voluntarily (Figure 1).

No statistically significant difference was found among the 3 groups with regard to demographic data, IELT and CIPE-5 baseline, shown in table 2.

**Table 2:** Demographic characteristics and IELT,Demographic characteristics and IELT,CIPE baseline (Mean±SD)

Group	TCMS	PEDT	TCMS plus PEDT	*P*-value
	(n=29)	(n=28)	(n=29)	
**Demographic characteristics**				
Age(year)	28.4 (3.8)	27.9 (3.3)	28.6 (3.9)	0.79
Height(cm)	171.6 (5.9)	172.5 (5.5)	171.3 (5.4)	0.70
Weight(kg)	67.0 (6.8)	66.3 (7.3)	66.5 (7.4)	0.93
BMI(kg/m^2^)	22.7 (1.6)	22.2 (1.6)	22.6 (1.6)	0.48
Course of disease(year)	4.5 (2.9)	4.3 (2.8)	4.6 (3.0)	0.93
**Baseline (pre-treatment)**				
IELT (min)	1.15(0.36)	1.16 (0.31)	1.13 (0.27)	0.87
CIPE-5 (score)	9.93 (2.98)	10.36 (3.09)	10.03 (2.98)	0.60

BMI: Body mass index;

IELT: Intravaginal ejaculation latency time;

CIPE-5: Chinese index of sexual function for premature ejaculation

Kruskal-Wallis analysis of variance was used to compare the median among each group.

### IELT, CIPE-5 change from baseline to the end of treatment.

IELT increased significantly from 1.15 min to 2.99 min in TCMS group (p< 0.01); from 1.16 min to 3.14 min in PEDT group (p< 0.01) and from 1.13 min to 4.36 min in TCMS plus PEDT group (p< 0.01). CIPE-5 scores elevated from 9.93 to 19.69 in TCMS group (p< 0.01), from 10.36 to 20.11 in PEDT group (p< 0.01) and from 10.03 to 23.14 in TCMS plus PEDT group (p< 0.01). Both TCMS and CIPE-5 in TCMS plus PEDT group after treatment was significantly higher than in TCMS (both p< 0.05) and PEDT group (both p< 0.05). (table 3)

**Table 3:** IELT and CIPE-5 levels before and after treatment in each group (Mean±SD).

Outcome Variable	TCMS	PEDT	TCMS plus PEDT	*P* a
**IELT (min)**				
Baseline	1.15 (0.36)	1.16 (0.31)	1.13 (0.27)	0.87
End point	2.99 (1.40)	3.14 (1.23)	4.36 (1.31)*#	< 0.01
*P*^b^	< 0.01	< 0.01	< 0.01	
**CIPE-5 (score)**				
Baseline	9.93(2.98)	10.36(3.09)	10.03(2.98)	0.60
End point	19.69(3.93)	20.11(3.83)	23.14(2.64)*#	< 0.01
*P* b	< 0.01	< 0.01	< 0.01	

a Kruskal-Wallis test was used to compare the median among each group.

b Kruskal-Wallis test was used to compare the median between the levels before and after treatment.

* p< 0.05 when compared with TCMS group.

# p<0.05 when compared with PEDT group

Abbreviations as in Table 2.

### Efficacy

**Figure 2:**
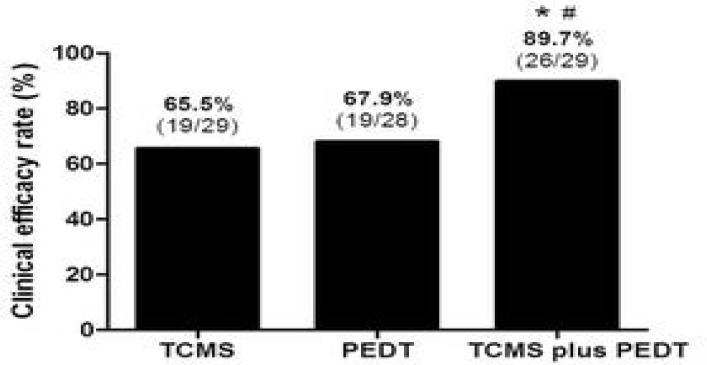
Clinical efficacy rate in each group. * Compared with TCMS group (p< 0.05); #compared with PEDTgroup (p< 0.05); No significant difference exists between TCMS and PEDT group

When the criteria of clinical efficacy was defined as the IELT ≥ 2 min and the CIPE-5 ≥18 scores after treatment, the number of eligible patients was 19, 19 and 26 in TCMS, PEDT and combination group, respectively. The efficacy in combination group (89.7%) was significantly higher than that in TCMS group (65.5%) and PEDT group (67.9%) (both p< 0.01), no significant difference existed between TCMS and PEDT group, as shown in figure 2.

### Side effects in each group.

Throughout the study, no serious systemic or local side effect occurred except that slight burning sensation presented by part of the patients, but this slight discomfort was tolerable.

## Discussion

PE is the most frequent male sexual dysfunction with a serious effect on the quality of life for both the patient and partner^20^,^26^. In this study, we employed IELT as the main measure for comparing different treatment options as it is the most universally accepted tool. Data from previous studies indicated that IELT measurement alone is not sufficient for accurately determining the PE status^27^,^28^, so another useful index CIPE-5 was employed for the evaluation of ejaculation control, sexual satisfaction of patients and partners, as well as personal distress. Employing a cutoff score of 18, the sensitivity of CIPE-5 was 97.60%, the specificity 94.74%^29^.

This present study showed that both IELT and CIPE-5 significantly increased in the three groups, whereas the increase in TCMS plus PEDT group was more than that in other two groups (Table 3). Additionally, clinical efficacy rate in TCMS plus PEDT group (89.7%) was also significantly higher than that in TCMS group (65.5%) and PEDT group (67.9%) (Figure 2). The results demonstrated that a combination of TCMS and PEDT therapy was more efficient for the treatment of PPE than TCMS or PEDT monotherapy.

All the patients were satisfied with their tolerance to the treatment in each group, no side effect was reported by the subjects in each group during the treatment course. It is worth mentioning that the combination therapy as well as the single TCMS therapy should not be used for the patients allergic to alcohol. Present therapies for the treatment of PE mainly include systemic pharmacological agents, topical agents and behavioral therapy, none of them seems to be ideal. Systemic pharmacological agents like selective serotonin reuptake inhibitors (SSRIs) and phosphodiesterase-5 (PDE-5) inhibitors all have systemic side effects, such as dry mouth, nausea, decreased libido, erectile dysfunction and so on^30^. Topical agents including severance secret-cream(SS-cream), dyclonine/alprostadil cream, lidocaine spray, prilocaine-lidocaine spray, all of them can reduce the sensitivity of the glans penis meanwhile the side effect cannot be ignored. The most common side effects of these agents' use are associated with localized irritation, including pain, burning, delayed ejaculation, loss of penile sensation^21^,^31^,^32^, wiping off before intercourse or condom use is necessary to avoid the side effect of decreased vaginal sensitivity of the partners. However, in our study, no serious systemic or local side effect occurred except that slight burning sensation presented on part of patients, but this slight discomfort was tolerable. Therefore, we believed that the self-made TCMS was safe and could obtain a good compliance.

Traditional herbs have long been used for the treatment of PE in China, India and several other countries. From the traditional Chinese medicine point of view, kidney yang failure is one of the main reasons for premature ejaculation, therefore the herbs with hot property such as *Radix Aconiti, Herba Asari, Syzygium aromaticum* and *Pericarpium Zanthoxyli* were selected to warm the kidney yang. *Ootheca Mantidis, Galla Chinensis, Fructus Rosae Laevigatae* and *Fructus Rubi* have the property of convergence, so we used them to prevent premature ejaculation. Modern researches show that *Radix Aconiti* has a wide range of therapeutic effects, active components are alkaloids such as aconitine, these alkaloids have pain-relieving effects, cardiotonic and vasodilator actions^14^,^33^,^34^, the vasodilator action may be induced by the increased yield of nitric oxide^14^, the latter has been detected as a neurotransmitter involved in the central and peripheral control of ejaculation, so *Radix Aconiti* may inhibit seminal emission by increasing the production of nitric oxide.

*Herba Asari* is rich in volatile oils, as the characteristic components, methyl eugenol and safrole may have local anesthetic and analgesic effects^35^. Studies show that *Pericarpium Zanthoxyli* has an obvious effect of local anesthesia and the main active ingredient is amide compounds, volatile oil. Water soluble matter of *Pericarpium Zanthoxyli* can reversibly block nerve impulse conduction and lower neural stem excitability of toad sciatic nerve, this may be the physiological basis of local anesthesia induced by *Pericarpium Zanthoxyli*^36^. *Syzygium aromaticum* has long been used in China and India to treat male sexual dysfunction, animal experiments showed that low dose of *Syzygium aromaticum* might have androgenic effect which could increase the motility of sperm and stimulate the secretory activities of epididymis and seminal vesicle^37^. The active ingredients of *Syzygium aromaticum* may also have ROCK-II inhibitory potential which could result in relaxation of corpus cavernosum, thus it has an effect on sexual dysfunction^12^. *Galla Chinensis* is rich in tannic acid which can lead to protein precipitation^38^. *Galla Chinensis* may treat PE through thickening skin and mucous membranes of the glans thus reducing its sensitivity.

Overall, the referring herbs may treat PE from different angles, both *Radix Herba Asari* and *Pericarpium Zanthoxyli* have local anesthesia effect, *Galla Chinensis* can lead to proteins precipitation, so they may treat PE by reducing the sensitivity of glans. *Radix Aconiti* has vasodilator action and *Syzygium aromaticum* can inhibit corpus cavernosum relax so as to promote penis hyperemia, *Radix Aconiti* may also inhibit the function of the sympathetic nervous system activity through increased NO and then inhibit ejaculation. *Syzygium aromaticum* may have androgenic effect. These herbs may play a role of superposition for the treatment of PE. There were also other desensitising agents which were promising for the treatment of PE. Dinsmore et al^39^ carried out a phase III, double-blind, placebo-controlled study and showed that PSD502 (three actuations of spray each containing 7.5 mg lidocaine and 2.5 mg prilocaine) improved ejaculatory latency, control and sexual satisfaction when applied topically 5 min before intercourse in men with PE. IELT in that study increased by 6.3-fold of baseline level after 3-month treatment. In another two double-blind, placebo-controlled, phase III study carried out by Carson et al^40^, PSD502 applied topically to the glans penis 5 minutes before intercourse also showed significantly improved ejaculatory latency, ejaculatory control, sexual satisfaction and distress, and no severe side effects occurred. The baseline levels of IELT in our study was much higher than that in the above studies, and the fold of increase for IELT was also different, this may be due to the duration of treatment and also the dose of treatment. Both the two therapies were promising for clinical use since they were effective and safe.

In this study, we used PEDT therapy supported by Weili Multifunctional Andrologic Disease Diagnosis And Treatment workstation (WLZZ-9999) instead of behavioral therapy completed by the patients and their sexual partners. The results showed that both PEDT and TCMS were effective for the treatment of PPE, however the combined therapy is far more effective than the mono-therapy. This study is only a preliminary exploration for the effect of self-made TCMS as well as combined TCMS with PEDT, we just provided an idea for the development of new therapeutic agent for the treatment of PPE, components in TCMS are complex and mechanism may be multifaceted, further research should be carried out to sieve the good from the bad and optimize the formula, deeply explore the mechanism of action.

## Conclusion

This study confirmed that the self-made TCMS was safe and effective, the combination of TCMS and PEDT therapy was more effective than the TCMS or PEDT mono-therapy. However, components in TCMS are complex and mechanism may be multifaceted. Further research should be carried out to clarify the active ingredients in TCMS and their interaction.
